# Attentional scope is reduced by Internet use: A behavior and ERP study

**DOI:** 10.1371/journal.pone.0198543

**Published:** 2018-06-08

**Authors:** Ming Peng, Xianke Chen, Qingbai Zhao, Zongkui Zhou

**Affiliations:** Key Laboratory of Adolescent Cyberpsychology and Behavior (CCNU), Ministry of Education; School of Psychology, Central China Normal University, Wuhan, China; Southwest University, CHINA

## Abstract

As a crucial living environment, the Internet shapes cognition. The Internet provides massive information that can be accessed quickly via hyperlinks, but the information is typically fragmentary and concrete rather than integrative. According to construal level theory, the processing of this concrete and fragmentary information, should reduce attentional scope. Two experiments were conducted to test this hypothesis. In Experiment 1, three groups of participants were asked to shop online, read magazines or have a rest respectively, and a divided attention Navon-letter task was employed to measure the attentional scope before and after the assigned activity. It was found that the difference between reaction times in response to local vs. global targets was decreased only after Internet use, while there was no decrease in either the reading or resting group. In Experiment 2, the same procedure was used, and EEG/ERP methods were used to record both behavioral response and neural activity. Results showed that before the assigned activity, there was no significant difference in N2 amplitude in response to local vs. global targets in any of the three groups; during the activity, the lower-alpha activity induced by Internet use was significantly lower than that induced by reading or resting; after the activity, correspondingly, a more negative N2 wave was induced by the global than local targets only in the Internet group, while there were no significant differences in the other groups. Consistent with construal level theory, the results suggest that when surfing the Internet, attentional scope is reduced, and this effect might continue after Internet activity.

## Introduction

According to evolutionary psychology, human cognition was shaped in specific environments [1, 2]. In order to adapt to these varying environments, humans developed many technologies, such as tools, language, and writing. These technologies in turn further affected human cognition [3]. Today, a recent technological invention, the Internet, also shapes our cognition. More than a simple tool, the Internet has developed into a crucial living environment.

The internet transmits the most information in our times which makes information acquisition an important activity on the Internet [4]. Importantly, the manner in which information is presented on the Internet is very different from how it is presented in life outside the Internet. For example, hyperlinks offer quick access to other documents and a vast store of knowledge that would not be readily available offline [5].

There is clear evidence to suggest that processing online information has a negative effect on cognition. Several studies have found that people’s browsing and scanning behaviors increased and their sustained attention decreased when reading online [3, 6, 7]. The Internet increases multitasking, leading to increased distractibility and impaired executive control abilities [8, 9]. Moreover, Carr [5] highlighted that hypertext environments, with increased processing demands, could reduce the cognitive resources available for deeper processing and memory consolidation. Construal level theory [10] is a useful model for understanding the cognitive processing of online and offline information. According to this model, the perception of psychological distance influences how people process certain types of information, and vice versa. For example, the concepts “tomorrow” and “next year” are perceived psychologically as being near and far; we use concrete thinking (a low level of mental construal) to process information that is perceived to be near, and abstract thinking (a high level of mental construal) to process information that is perceived to be far [10, 11]. Abstract information enlarges the scope of cognition, whereas concrete information reduces the scope of cognition [10, 11]. Specific to the situation of the Internet, the presentation of information online (e.g. a vast amount of knowledge, a hyperlinked environment) increases browsing and scanning behaviors [3, 6], making it difficult to process information deeply [5]. Therefore, our cognitive scope would zoom in (would decrease) when using the Internet because of the predominance of concrete information, and there would be little integrative thinking in this situation.

Cognitive scope can vary at attentional or conceptual levels [12]. In this study, we focused on the attentional level. We compared the attentional scope of three groups of participants before, during and after they engaged in an assigned task (shop online, read magazines, or rest). Before and after the assigned task, participants were asked to judge Navon-type compound letters, each of which was a large letter composed of several small letters [13]. There are two common types of Navon-letter tasks, one being a divided attention task and the other a selective attention task [14]. We used a divided attention task in the current study. In this type of task, subjects respond to targets that appear at the global level (large letter) or at the local level (small letter) with equal probability within the same block of trials. The divided attention task is often used to examine the processing of global/local features of hierarchical stimuli [14, 15]. It has been assumed that the identification of a local target (small letter) requires focused attention on local elements, whereas the identification of a global target (large letter) requires an enlarged scope of visual attention [16]. If participants show faster reaction time in identifying the global letter than the local letter, they are showing a global processing style; otherwise they are showing a local processing style.

In addition to the behavior assessment, we also used EEG/ERP data as a measure of attentional scope. Studies on the topic of attention have shown that ERP components reliably reflect differential attentional scope. The N2 component, a negative-going wave that peaks 200-350ms post-stimulus over frontal-central scalp sites, has been found to index cognitive control, encompassing response inhibition, response conflict, and error monitoring [17, 18, 19]. Heinze and Münte [20], in their first study, identified N2 as a sign of early global/local target perception. Later, studies found a smaller N2 component induced by global stimuli than local stimuli in global-local processing [18, 21, 22]. In a Navon-letter task, participants should inhibit the influence of local letters on the global letter judgment and vice versa, and the requirement of cognitive control should modulate the amplitude of the N2 component. Therefore, the smaller N2 component induced by global vs. local stimuli means a global priority [18, 21, 22]. In the current study, if the attentional scope was reduced by Internet use, participants would process local targets relatively more easily and react to global targets with relatively more difficulty. This would lead to a larger N2 component induced by global letters.

However, the changes in both reaction times and the N2 component in the divided attention task would be indirect indices of the possible influence of Internet use on attentional scope, because they are measured after rather than during Internet use. Comparatively, the neural activities when participants are engaged in the assigned task might give more direct evidence for the reduced attentional scope in the Internet group. Previous studies reported that lower-alpha (8-10Hz) activity was associated with change in alertness, which is one of the basic functions of attention [23, 24]. Because it is more likely to reflect general demands rather than specific task requirements [25], lower-alpha activity might be interpreted as defocused attention [26]. In other words, a decrease in lower-alpha activity during Internet use might suggest an increase in alertness or a focused state, indicating a reduced attentional scope [27].

We conducted two experiments to test the effect of Internet use on attentional scope. In both experiments, participants engaged in one of three types of activity (using the Internet, reading magazines, or resting) and were then asked to engage in a Navon-letter task. In the Internet group, we allowed participants to find the goods that he/she want to buy. The shopping behavior on Internet was a highly frequent behavior, which was very similar with the other information acquisition behaviors, like reading news, or checking WeChat moment. In the reading magazines group, we also asked participants to find the goods that he/she wanted to buy. Therefore we set a matched online and offline task.

Experiment 1 was a behavior study in which attention was assessed in terms of reaction times in response to global and local stimuli in the task, measured before and after the assigned activity. Experiment 2 also assessed reaction times, but was essentially an EEG study in which attention was assessed in terms of N2 before and after the assigned activity and lower-alpha activity during the assigned activity. In the experiments, after the assigned activity, participants were asked to evaluate their emotional state during the assigned activity to rule out the influence of emotional state on the results. In both experiments, the hypothesis was that a greater reduction in attentional scope would be seen in the Internet use group than in the reading and resting groups.

## Experiment 1

### Method

#### Experimental design

A 3×2×2 mixed experimental design was used with the main effects being priming task (Internet group, reading magazine group, resting group), test session (pretest, posttest) and target letter type (global, local).

#### Participants

This study and Study 2 to be reported next were carried out in accordance with the ethics principles of the Declaration of Helsinki. All participants signed an informed consent form for the experiment. The informed consent form and the two studies were reviewed and approved by the ethics committee of the Faculty of Psychology, Central China Normal University, China.

181 students were selected in Experiment 1. Their mean age was 19.79 years (SD = 1.47). They were randomly divided into three groups, in which 62 students (28 males) engaged in Internet group, 62students (20 males) engaged in resting group, and 57 students (20 males) engaged in reading magazine group.

#### Materials

A 20-item measure of Young’s Internet Addiction Test (IAT) [28] was used to examine whether or not there were significant differences among the IAT scores of the three groups. The level was evaluated on a 5-point scale scored from 1 (never) to 5 (always). The score higher than 80 reflects Internet addiction [29].

The stimuli used in divided attention task were global letters made up of local letters in a 5×5 matrix. A global letter measured 4.7 cm height and 4.1 cm width, while a local letter measured 0.7 cm height and 0.6 cm width. The stimuli contained a target either at the global level (i.e., global/local letters were E/F, E/L, E/T, or H/F, H/L, H/T) or at the local level (i.e., global/local letters were F/E, L/E, T/E, or F/H, L/H, T/H), resulting in twelve stimuli.

#### Procedure

Each participant was seated in a comfortable chair facing a computer screen at a distance of 60 cm. Stimuli were presented on a 17-inch CRT monitor through Inquisit 3.0 software (Millisecond Software Inc., Seattle, Washington, USA).

After participants came into the lab, they were asked to perform a divided attention task as a pretest. Then they were arranged into one of priming tasks. The Internet group was asked to go shopping in the online shopping site (add the items to the shopping cart), the reading group was asked to read some magazines and find something that they wanted to buy (write down the goods in a sheet), while the resting group was asked to sit silently and to keep awake. The priming task lasted 15 min. After that, they performed the divided attention task as a post test. At last, they were required to complete demographic information, questionnaires of Young Internet addiction test, and praised the emotional valence and arousal induced by the priming task. The Self-Assessment Manikin (SAM) rating scales [30] for pleasure and arousal were used and were on display for the rating period only. Valence ratings ranged from 1 (very unhappy) to 9 (very happy) and arousal ratings ranged from 1 (very calm) to 9 (very excited).

In the divided attention task, at the beginning of each trial, a fixation cross was displayed for 500 ms to signal that a trial was about to begin. The target display was presented immediately after the fixation display and remained on the screen until participants identified the target letter by pressing either “F” or “J” key (corresponding to left and right forefinger). Half the participants pressed the “F” key with left forefinger to indicate an “E” letter and “J” key with right forefinger to indicate an “H” letter. The other half of the participants used the reverse stimulus-response mapping. If wrong, a red exclamation mark presented. After that a blank presented 1000 ms as an interval. Participants received 4 practice trials (make sure they remembered the answers), then followed by 20 experimental trials.

### Results

#### Young IAT scores

The average score in Young IAT was 48.75 (SD = 10.97). There was no significant difference among three groups on the test, *F* (2, 178) = 1.07, *p*> 0.3.

#### Emotion evaluation results

We used priming task (Internet group, reading magazine group, resting group) as independent variable and did one-way ANOVA on emotional valence and emotional arousal. We found significant effect on emotional valence *F* (2, 179) = 7.09, *p* < 0.01, *η*_*p*_^*2*^ = 0.07, and on emotional arousal *F* (2, 179) = 9.74, *p* < 0.001, *η*_*p*_^*2*^ = 0.09. Further multiple comparisons showed that in emotional valence, there was a significant difference between Internet group (M = 6.61, SD = 1.41) and resting group (M = 5.79, SD = 1.42) (*p* < 0.01), and between reading magazine group (M = 6.59, SD = 1.31) and resting group (*p* < 0.01), but no significant difference between Internet group and reading magazine group. In emotional arousal, there was a significant difference between Internet group (M = 5.13, SD = 2.03) and resting group (M = 3.65, SD = 1.80) (*p* < 0.001), and between reading magazine group (M = 4.58, SD = 1.83) and resting group (*p* < 0.01), but no significant difference between Internet group and reading magazine group.

#### Reaction times results

**Fig 1:**
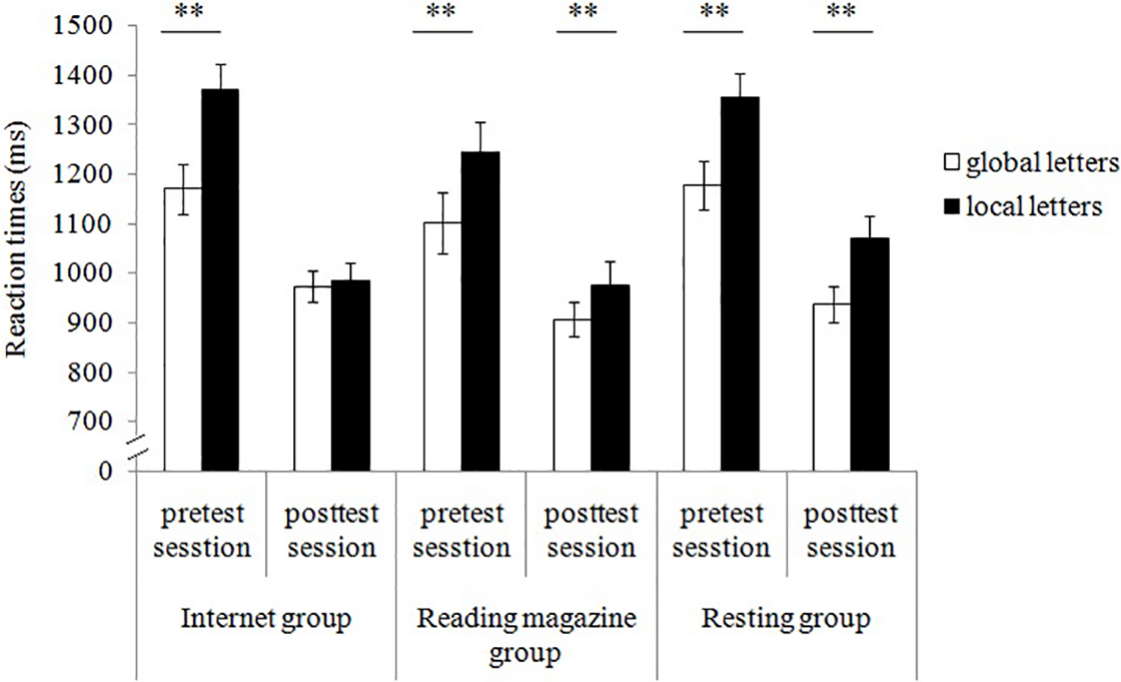
Reaction times for global and local letters during pretest and posttest session in each group.

One female and one male in resting group were deleted because of too much wrong reaction in one or two groups. Wrong reactions and correct reaction times exceeding the mean by three standard deviations were excluded from data analysis, resulting in the removal of 6.3% of the trials. ANOVAs of RTs showed significant main effects of test session *F* (1, 176) = 130.63, *p* < 0.001, *η*_*p*_^*2*^ = 0.43, and target letter type *F* (1, 176) = 71.67, *p* < 0.001, *η*_*p*_^*2*^ = 0.29. It also showed significant interaction effects between test session and priming task *F* (2, 176) = 3.40, *p* < 0.05, *η*_*p*_^*2*^ = 0.04, test session and target letter type *F* (1, 176) = 14.19, *p* < 0.001, *η*_*p*_^*2*^ = 0.08, and a significant interaction effect among three factors *F* (2, 176) = 4.13, *p* < 0.05, *η*_*p*_^*2*^ = 0.05. In the pretest session, there was no significant interaction between target letter type and priming task *F* (2, 176) = 1.15, *p*>0.05. Faster reaction for global letters than local letters was found in Internet group *F* (1,178) = 26.78, *p* < 0.001, reading magazine group *F* (1,178) = 8.57, *p* < 0.01, and resting group *F* (1,178) = 19.51, *p* < 0.001. In the posttest session, there was a significant interaction between target letter type and priming task *F* (2, 176) = 4.64, *p* <0 .05. Faster reaction for global letters than local letters was found in reading magazine group *F* (1,178) = 7.71, *p* < 0.01, and resting group *F* (1,178) = 21.11, *p* < 0.001, but not in Internet group *F* (1,178) = 0.17, *p* > 0.8 (Fig 1).

### Discussion

Results of Experiment 1 showed that in the pretest, reactions to global letters were faster than those to local letters in all three groups; in the posttest, reactions to global letters were still faster in both the reading magazine and resting group, while the difference between global letters and local letters was not significant in the Internet group. These results suggest that the precedence of global processing was not changed in either the reading or resting group, while it was eliminated after shopping online in the Internet group. In other words, by assessing reaction time, Experiment 1 verified that Internet use reduced participants’ attentional scope.

We also found the reaction times were significant different between pretest and posttest, which suggested that as the participants became familiar with the procedure and had the experience on the judgment, the reaction times would decrease.

However, reaction time data provide only an indirect index of the possible influence of Internet use on attentional scope. Therefore, in Experiment 2, the EEG/ERP technique was employed. This method would provide independent indices of neural activity in the different priming conditions, potentially confirming the conclusions based on behavioral data. More importantly, the EEG/ERP technique could record neural activity during the priming task, offering more direct evidence of a reduction in attentional scope in the Internet group.

## Experiment 2

### Method

#### Participants

There were 60 students participated in Experiment 2, who were averagely divided into the Internet, reading magazine and resting group, with 10 males in each group. Their mean age was 19.57 years (SD = 1.54).

#### Materials

The stimuli used were same with that in Experiment 1.

#### Procedure

Following the application of the electrodes, each participant was seated in a comfortable chair facing a computer screen at a distance of 60 cm. Stimuli were presented on a 17-inch CRT monitor through E-Prime software (Psychological Software Inc., Sharpsburg, Pennsylvania, USA).

The procedure was same with experiment 1. Participants received 4 practice trials and make sure they remember the answer, then followed by 80 experimental trials.

#### Electrophysiological recording and analysis

Brain electrical activity was recorded from 64 scalp sites using tin electrodes mounted in an elastic cap (Brain Products, Gilching, Germany). The reference electrode was placed at FCz. The electrooculogram (EOG) was recorded with electrodes placed under the right pupil (VEOG) and at the outer corners of the right eye (HEOG). All interelectrode impedances were maintained below 20 KΩ. The EEGs were amplified using a 0.05–100 Hz bandpass filter and continuously sampled at 250 Hz/channel for off-line analysis.

EEG data were re-referenced to left and right ear mastoids (average signals of Tp9 and Tp10) before further analysis. A low-pass filter with a bandpass of 0.1–30 Hz was adopted to remove the high-frequency noise and the Independent Component Analysis was used to reject the eye movement artifacts (blinks and eye movements) during the off-line analysis. Meanwhile, the artifact induced by blinks, eye movements, excessive muscle activities were also eliminated offline (voltage exceed ±100 μV in any channel). Then stimulus-locked analysis were applied to segment and average the ERPs elicited by the global and local processing, in which only the trials with correct response were included. In the stimulus-locked analysis, the event was time-locked at 0-1000ms after the Navon-letter presented on the screen with the baseline pre-stimulus 200ms.

The focus of our analysis was the difference between the ERPs elicited by global processing and local processing. The difference waves were obtained by subtracting the averaged ERP of global processing from the averaged ERP of local processing. And this difference was prominent over the fronto-central scalp regions during 250-400ms after stimulus onset. Thus, the following 8 electrode sites (F5, FC5, C5, CP5, F6, FC6, C6, CP6) were chosen for three-way repeated measures ANOVA to analyze the mean amplitudes of N250-400. The ANOVA factors were priming task (Internet group, reading magazine group, resting group), test session (pretest, posttest) and target letter type (global, local).

Spectral analysis was also performed with EEG data. In this analysis, 30-s time intervals between blocks were used as reference interval while 15-min time intervals during task were used as task interval. For reference interval, due to “settling” and “ending” artifact, the first and last second were discarded, and for task interval, the first and last minute were discarded. For all the intervals, artifact-free epoch 4s were subjected to the fast Fourier transformation (FFT) to calculate EEG band power (μV^2^) within a lower (8-10Hz) alpha frequency band. Task related power (TRP) changes were computed using the following formula:

$$TRP_{i}=\log(Power_{i\;task})-\log(Power_{i\;reference})$$

The power during the reference interval was log-transformed and subtracted from the log-transformed power during the task interval. Thus, negative TRP values reflected decreases in alpha band power from the reference to the task, whereas positive TRP values reflected task-related increases. The electrode sites chosen to analyze were the same as ERP analysis. Separate one-way ANOVA for lower-alpha were computed with one between-subject factor that is priming task.

### Results

#### Young IAT scores

The average score in the Young IAT was 47.58 (SD = 12.02). There was no significant difference among three groups on Young IAT scores, *F* (2, 59) = 0.475, *p* >0.6.

#### Emotion evaluation results

We used priming task (Internet group, reading magazine group, resting group) as independent variable and did one-way ANOVA on emotional valence and emotional arousal. We found a significant main effect on emotional valence *F* (2, 59) = 3.69, *p* < 0.05, *η*_*p*_^*2*^ = 0.12, and on emotional arousal *F* (2, 59) = 3.25, *p* < 0.05, *η*_*p*_^*2*^ = 0.10. Further multiple comparisons showed that in emotional valence, there was a significant difference between Internet group (M = 6.70, SD = 1.26) and resting group (M = 5.90, SD = 1.45) (*p* < 0.05), between reading magazine group (M = 6.95, SD = 0.99) and resting group (*p* < 0.05), but no significant difference between Internet group and reading magazine group. In emotional arousal, there was a significant difference between Internet group (M = 4.10, SD = 2.10) and resting group (M = 2.85, SD = 1.63) (*p* < 0.05), between reading magazine group (M = 4.25, SD = 1.65) and resting group (*p* < 0.05), but no significant difference between Internet group and reading magazine group.

#### Reaction times

Wrong reactions and correct reaction times exceeding the mean by three standard deviations were excluded from data analysis, resulting in the removal of 5.10% of the trials. ANOVAs of RTs showed significant main effects of test session *F* (1, 57) = 81.04, *p* < 0.001, *η*_*p*_^*2*^ = 0.59, and target letter type *F* (1, 57) = 8.95, *p* < 0.01, *η*_*p*_^*2*^ = 0.14. It also showed a significant interaction effect between test session and target letter type *F* (1, 57) = 5.31, *p* < 0.05, *η*_*p*_^*2*^ = 0.09, and a significant interaction effect between priming task and target letter type *F* (2, 57) = 5.43, *p* < 0.01, *η*_*p*_^*2*^ = 0.16. Faster reaction for global letters than local letters was found in the pretest session, *F* (1, 59) = 9.23, *p* < 0.01, but not in the posttest session, *F* (1, 59) = 2.80, *p* > 0.05. Significant faster reaction for global letters than local letters was only found in the reading magazine group *F* (1, 57) = 18.20, *p* < 0.001, but not in resting group *F* (1, 57) = 0.66, *p* > 0.4, and Internet group *F* (1, 57) = 0.01, *p* > 0.9. There was no significant interaction effect among three factors.

#### Electrophysiological data (ERP data)

**Fig 2:**
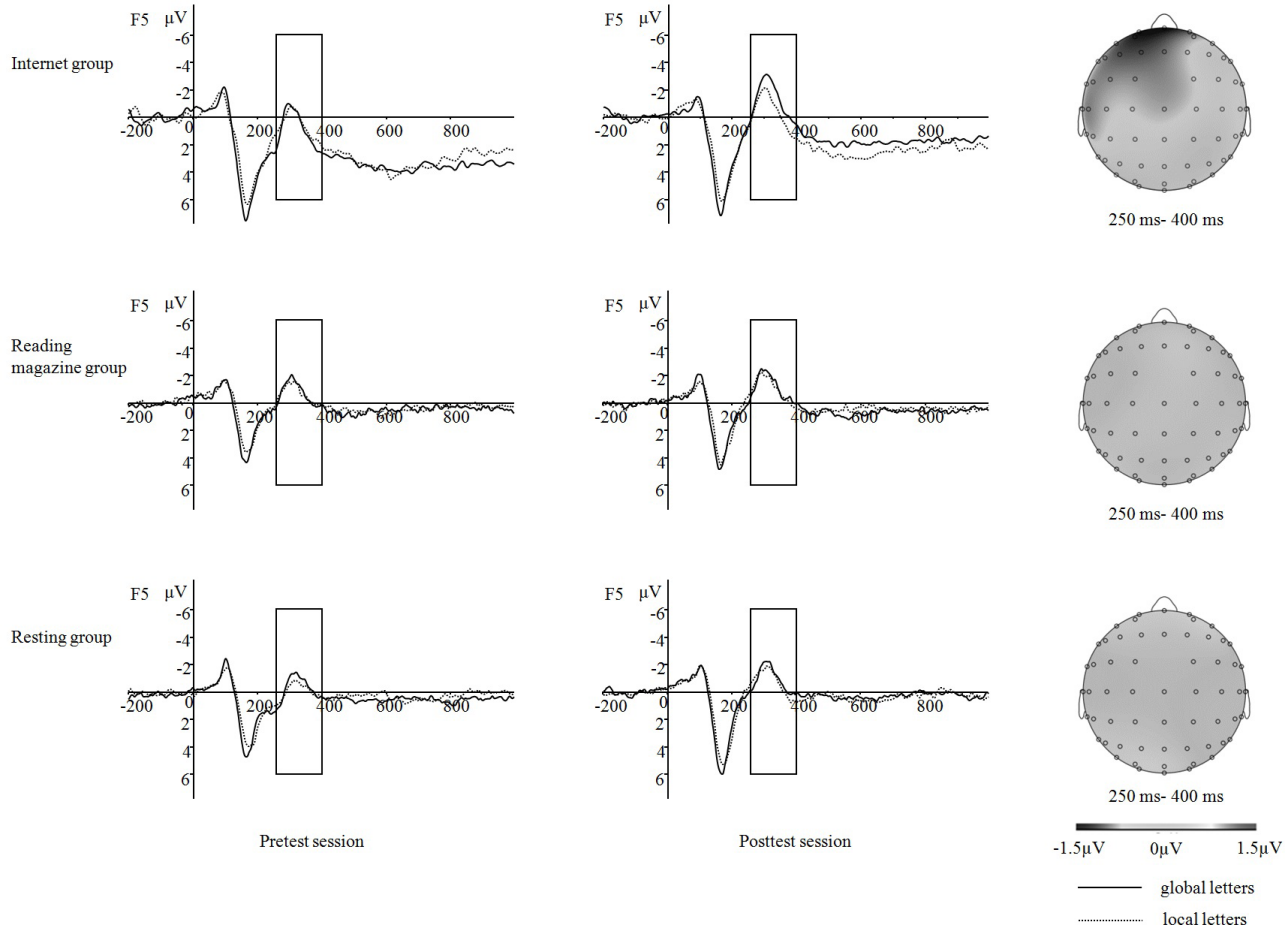
ERP waveforms and topography in each group. ERP waveforms on F5 site for global letters and local letters during pretest and posttest session in each group, and topography of the effect of time on task on the difference in N2 amplitude between the pretest and posttest session on target letter type effect (global letters minus local letters) in each group.

Three-factors repeated measures ANOVA of N2 amplitudes showed the significant main effects of test session, *F* (1,57) = 22.57, *p* < 0.001, *η*_*p*_^*2*^ = 0.28, and target letter type *F* (1, 57) = 4.53, *p* < 0.05, *η*_*p*_^*2*^ = 0.07. It also showed a significant interaction effects between priming task and test session, *F* (2, 57) = 10.71, *p* < 0.001, *η*_*p*_^*2*^ = 0.27, and a significant interaction effect among three factors, *F* (2, 57) = 3.21, *p* < 0.05, *η*_*p*_^*2*^ = 0.10. The simple effect analysis indicated that in the pretest session, no effect was significant, while in the posttest session, the main effect of target letter type *F* (1, 57) = 5.73, *p* < 0.05, and the interaction effect between target letter type and priming type *F* (2, 57) = 3.72, *p* < 0.05 were significant. In the posttest session, the ERP elicited by the global letters was more negative than that by the local letters in Internet group *F* (1, 57) = 12.83, *p* < 0.001, while there were no target letter type differences in resting group and reading magazines group (Fig 2).

#### Task-related power during different tasks

**Fig 3:**
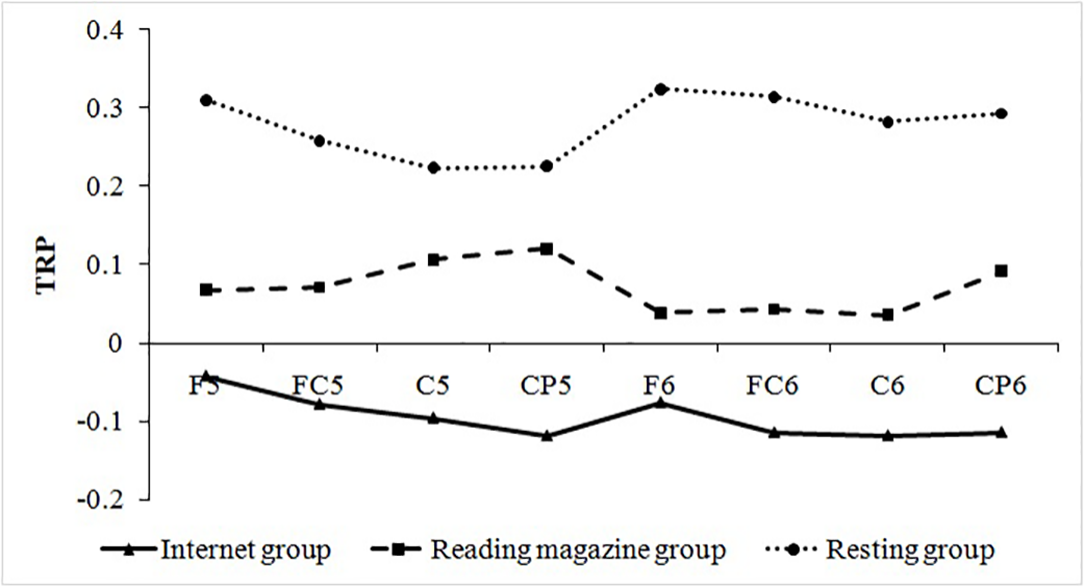
Task related power (TRP) changes during priming task in each group.

In lower-alpha band, one-way ANOVA revealed a significant effect of priming task, *F* (2, 57) = 10.61, *p* < 0.001, *η*_*p*_^*2*^ = 0.271. Subsequent post-hoc analysis (Student Newman-Keuls) revealed that subjects in Internet group got the lowest TRP scores of three groups (M = -0.09, SD = 0.21), and subjects in reading magazines group (M = 0.07, SD = 0.25) got lower TRP scores than resting group (M = 0.28, SD = 0.30) (Fig 3).

### Discussion

Experiment 1 found a significant interaction among the three variables of interest (i.e. priming type, test session, and target letter type) in predicting reaction time. This interaction indicated that the precedence of global perceptive processing disappeared after priming with an Internet task, reflecting a reduction of attentional scope. By contrast, the interaction among these three variables in predicting reaction time was not significant in Experiment 2. This finding will be considered in more detail in the General Discussion section.

The ERP results in Experiment 2 were similar to the reaction time results in Experiment 1 in documenting the expected interaction effect. The ERP results showed that, in the pretest session, there was no significant difference in N2 amplitude induced by global vs. local letters, in any of the three groups. In the posttest session, a similar lack of difference was seen in the reading and resting groups, but in the Internet group, the N2 amplitude induced by global letters was larger than that induced by local letters. As discussed in the Introduction, larger amplitude of N2 might reflect greater cognitive control. Therefore, in the posttest the larger N2 amplitude induced by global vs. local letters in the Internet group suggests more cognitive control, requiring more cognitive resources, when processing the global letters. This might result from the reduction of attentional scope by Internet priming.

Furthermore, we found a more reduced lower-alpha activity in the Internet group than in the other two groups. Because low-alpha activity is associated with defocused attention [27], decreased lower-alpha activity might reflect a concentrated state and reduced attentional scope [23]. Consistent with our expectation, the low-alpha activity in the Internet group was lower than in the reading and resting groups. This suggests that participants’ attention was more concentrated in the Internet situation than in the reading and resting conditions, leading to a reduced attentional scope.

## General discussion

Our cognition is modulated to adapt in various new situations. The Internet is quite a new environment that is significantly different from offline situations. According to construal level theory, it can be inferred that some characteristics of the Internet situation (e.g., the way that information is presented) would lead to a reduction in attentional scope. Therefore, we conducted a behavior study and an EEG/ERP study to test whether attentional scope was decreased in the context of Internet use. Participants were assigned to one of three priming tasks: online shopping, reading magazines, or resting. The groups were compared on reaction times and N2 amplitudes when participants did divided attention task before and after the priming task, as well as lower-alpha activity in the course of the priming task. Results of reaction time data provided partial support for the hypothesis; ERP data showed strong support, suggesting that Internet use reduced attentional scope.

The results showed that before the priming task, participants in the three groups showed a similar mode of global-local processing, in both Experiment 1 (reaction time data) and in Experiment 2 (ERP data). However, after the priming task, participants in the magazine reading group and the resting group showed a mode of global-local processing that was similar to pretest, whereas participants in the Internet group showed relatively more difficulties in processing global letters compared with pretest. Furthermore, compared with the other two groups, a more decreased lower-alpha activity was found during the Internet priming task. These results supported the hypothesis that Internet use would decrease attentional scope.

The presentation of information is different on the Internet than in books or magazines, and this type of presentation may reduce attentional scope. Because the content of books and magazines is relatively fixed and limited, people are used to thinking about the information being presented rather than immediately searching for new information. However, on the Internet, continuous information is afforded by hyperlinks, and people usually look through the information quickly and then search for more information, rather than think closely about the original information [6, 7]. This processing of Internet information is a low level process because of the lack of synthesis. According to construal level theory [10], this kind of processing decreases the individual’s attentional scope.

In addition to being affected by presentation style, attentional scope is affected by affective state [12, 31, 32, 33]. Some dimensions of affective states are valence (which is the positive-to-negative evaluation of the subjectively experienced state), arousal (which is the psychophysical state in response to a stimulus), and motivational intensity (which is the strength of the urge to approach or withdraw from a stimulus)[12]. Earlier studies have shown that positive affective states broaden attentional scope, whereas negative affective states narrow attentional scope [34, 35, 36]. Motivational intensity has also been considered in recent studies on the effect of emotion on attentional scope[12].

In the current study, participants were asked to evaluate emotional valence and emotional arousal when they were engaged in the priming tasks. Results showed that the Internet group and magazine reading group showed significantly greater emotional valence and arousal than the resting group, but there was no significant difference between the Internet group and the magazine reading group. Because the current study did not use stimuli that have typically been used in former studies to modulate motivational intensity [12], such as monetary incentive or appetitive stimuli (such as desserts), motivational intensity would not have differed among the three groups. Therefore, emotional state was not a factor that changed attentional scope in the Internet group.

There were some results that were inconsistent with our expectations. First, unlike in Experiment 1, we did not find a significant interaction among the three variations (i.e. priming type, test session, and target letter type) in the reaction times in Experiment 2. This might be because the number of trials was larger in the ERP study than in the behavior study, which gave the participants a chance to practice, thus reducing the effect. Alternatively, Experiment 2 was an ERP experiment, which took more time than the behavioral study because of the preparation for EEG data collection. This might make participants tired, which would affect the behavioral performance. A previous ERP study also did not observe different reaction times in response to global vs. local stimuli [22].

Second, in some previous studies, a left hemisphere advantage has been reported for local processing and a right hemisphere advantage for global processing [17, 18]. However, we did not find a hemispheric difference in processing global and local letters. One possible reason was that the stimuli in this study were presented in the middle of the screen; the visual field presentation was not modulated. The lack of modulation could potentially lead to the disappearance of any hemisphere difference [36].

Third, the participants in the study did not differed on the IAT scores across three conditions. Future studies can choose the participants with and without the internet addictions in advance to investigate whether their effects will differ on different experiment groups.

## Conclusion

In this study, we investigated whether attentional scope would be influenced by Internet use. Experiment 1 assessed behavior and Experiment 2 assessed behavior as well as ERP/EEG responses. There was partial evidence that after Internet use, there was no longer a difference in reaction times in response to global letters compared to local letters in a Navon-letter task. The results were clearer based on the N2 amplitude data, which showed a larger response to global letters compared to local letters in the Internet group (but not the reading or resting groups). More importantly, the lower-alpha activity was lower during Internet activity than during magazine reading and resting. In all, using the Internet reduced people’s attentional scope.

## Supporting information

S1 File The behavioral data for experiment 1. (XLS)

S2 File The behavioral and ERP data for experiment 2. (XLS)
